# Determinants of Creativity in Migrant versus Urban Children: A Case Study in China

**DOI:** 10.3390/children11070802

**Published:** 2024-06-29

**Authors:** Yiqi Dai, Shunan Chen, Fengqian Mao, Junfang Xu

**Affiliations:** 1 WLSA Shanghai Academy, Shanghai 200433, China; cathydai0227@hotmail.com; 2 School of Pharmaceutical Sciences, Wenzhou Medical University, Wenzhou 325035, China; 3 School of Public Health, Second Affiliated Hospital, Zhejiang University School of Medicine, Hangzhou 310009, China

**Keywords:** creativity, migrant children, urban children, China

## Abstract

**Objective:** This study examines the current status and variations in creativity between migrant and urban children, exploring the influencing factors affecting creativity. **Methods:** We selected children from local households in Hangzhou City and non-local migrant households as participants. Their basic demographic information and creative tendencies were assessed using the Children’s Basic Situation Questionnaire and Williams’ Creativity Tendency Measurement Scale, respectively. A multi-model regression analysis was conducted to analyze factors influencing creativity. **Results:** This study included 1047 children. Significant differences were observed between urban and migrant children regarding age, family type, number of siblings, parental education, parental presence at home, parental guidance in learning, experience of changing schools, having their own room, and academic performance. In addition, migrant children exhibited significantly lower creativity levels compared to urban children. The multi-model regression analysis showed that migrant status, a good parent–child relationship, having parents who often guide learning, having their own room, and excellent academic performance significantly influenced children’s creativity. **Conclusions:** Migrant children display lower levels of creativity than their urban counterparts, with notable differences across several factors.

## 1. Introduction

The term creativity was coined by Guilford in 1950 and refers to the ability to propose and produce work products that are new and applicable [1]. Childhood is a critical period for the development of creativity, which plays an important role in children’s psychological development and is positively correlated with the subsequent adaptation, development, growth, and learning of the individual, and may also be a predictor of creativity in adulthood [2,3]. Therefore, children’s creativity has always been a hot area in educational research and practice, and the importance attached to children’s creativity in our country is increasing day by day [4].

In recent years, the number of migrant children has been increasing year by year, and all sectors of society have begun to pay attention to the education of migrant children. In studies comparing urban and rural children, there were differences in creative thinking and personality between urban and rural children, with urban children performing better on measures of creativity [5,6,7]. A study comparing creativity between urban, migrant, and rural children in China showed that migrant children scored significantly higher than rural children but lower than urban children [8]. There are also studies showing that urban children are more favorably disposed to creativity than rural and migrant children, but there are no significant differences between rural and migrant children [9]. Other studies have also shown significant differences in the creative thinking skills of urban, migrant, and rural children, with migrant children having significantly higher levels of creativity than their rural counterparts [10]. After comparing the creativity of mobile gifted children and urban gifted children, it was found that there was a significant difference in their creativity [11,12]. A comparison of 15 positive psychological qualities between migrant and urban children showed that urban children were significantly more creative than migrant children [13]. In addition, a study exploring the current state of psychological development of migrant children from both cognitive and personality perspectives found that the level of creativity of migrant children did not differ from that of urban children but was significantly higher than that of rural children [14]. The results of each study are not identical, so this study further explores the current status and differences in creativity between migrant and urban children.

Sternberg believes that everyone is creative, just to a different degree, and that creativity can be explored and nurtured [15]. Research into the factors influencing children’s creativity is an important prerequisite for training and developing creativity. Some studies have identified family self-esteem, classroom environment, openness, intellectual environment, and personal traits as factors influencing creative thinking [16]. In addition, studies have shown that socio-economic status, emotions, art education, play, etc., have an impact on children’s creativity [4,17,18,19]. A summary of the extensive literature outlines the factors influencing creativity as genetic factors, family factors, school factors, and personality qualities [20,21]. Compared with urban children, migrant children—as one of the vulnerable groups in the city—inevitably face many difficulties in the process of living in the city, such as problems with adapting to life, behavioral habits, learning problems, mental health problems, etc., and, thus, their creativity may be inhibited or even reduced [22]. This study will discuss the impact on creativity from multiple perspectives and identify significant influences.

Much of the current research on migrant and urban children has focussed on social development and mental health issues, with very little research focussing specifically on creativity, and most of the studies are older; however, the times are rapidly advancing and changing, and the results of the research on creativity may vary [23]. Therefore, this study discusses children’s creativity based on the present time and hypothesizes that the level of creativity of migrant children is lower than that of urban children and that household registration, family, and school factors are responsible for this difference.

By comparing the creativity of migrant and urban children, it is possible to study an important environmental variable in children’s development and provide valid evidence of its potential impact. The assessment of creativity is a prerequisite for fostering creativity, and this study not only understands and grasps the characteristics of children’s creativity development but also provides a theoretical basis for the development and cultivation of children’s creativity in the future, which is of practical significance for bringing high-quality education to the migrant population.

## 2. Data and Methods

### 2.1. Participants

In this study, the sample of urban children came from fourth-, fifth-, and sixth-grade students in a public school in Linping District, Hangzhou City, Zhejiang Province, where the enrollment requirements were local household registration and residence in the district for more than one year, and the sample of migrant children came from the same grades in a migrant children’s school in the same area, which was attended by children of migrant workers. The inclusion criteria for urban children were (1) children under the age of 18 in Hangzhou, Zhejiang Province; (2) children’s household registration in their current place of residence; and (3) children who had lived and studied in their current place of residence for a long time and had no experience of migration. The selection criteria for migrant children were (1) children under 18 years of age in Hangzhou, Zhejiang Province; (2) children whose household registration was not their current place of residence; and (3) children who had lived and studied in their current place of residence for at least half a year. A total of 1143 questionnaires were distributed, and 1047 valid responses were collected, yielding an effective response rate of 91.6%. Of these, 427 valid questionnaires were from migrant children, with an effective response rate of 92.2%, and 620 valid questionnaires were from urban children, with an effective response rate of 91.2%.

### 2.2. Instruments

First, demographic information of participants about the background and migration status of migrant and urban children were collected by 12 questions on four dimensions: household registration status, age and gender, family environment, and school environment. Second, an adapted Chinese version of Williams’ Creative Inclinations Test [24], designed to assess the creativity of children, using a 3-point rating scale across 50 questions including 42 positive and 8 negative items was used. It contains four dimensions of creativity: adventurousness (11 questions), curiosity (14 questions), imagination (13 questions), and willingness to challenge (12 questions). It is one of the most widely used instruments for measuring creativity. Participants rate each item on a scale of 1 to 3, where 3 points indicate full agreement (or non-agreement for negative items), 2 points indicate partial agreement (or non-agreement for negative items), and 1 point indicates full disagreement (or agreement for negative items). A higher score in a dimension suggests a stronger presence of that characteristic; similarly, a higher aggregate score across all dimensions indicates greater creativity. The Cronbach alpha coefficient for the sample of this study was 0.838, indicating that the questionnaire had high internal consistency.

### 2.3. Procedures

The research was conducted from November to December 2023. The questionnaires were administered in a centralized manner in classrooms to children who agreed to participate in this study, organized on-site by the researcher with the cooperation of the class teachers. Informed consent was obtained from the participating children and their parents.

### 2.4. Statistical Analysis

Firstly, the demographic characteristics of migrant and urban children were analyzed to observe the differences between the two groups through *t*-test and chi-square analysis. Second, for hypothesis one of this study, a *t*-test was used to compare whether there was a difference in the mean of the four dimensions of creativity and the total score between the two groups. Third, to address hypothesis two of this study, a multi-model regression analysis was conducted based on the four types of variables included in the Basic Information Questionnaire for Children (household registration status, age and gender, home environment, and school environment) to assess the influence of each factor on the creativity tendency of all students.

The dependent variable in the regression analysis was the total creativity score and the independent variables were the variables in the “Questionnaire on Basic Information of Children”. First, we regressed the total creativity score for child migrant status to obtain a baseline effect, where the coefficient showed the difference in creativity between migrant and urban children. In the second model, socio-demographic variables such as age and gender were included. The third model included variables related to parental/family structure, while the fourth model introduced variables related to academic performance and transferring to another school. This approach was adopted to examine the influence of these variables on the relationship between migrant status and children’s creativity.

## 3. Results

### 3.1. Comparison of General Socio-Demographic Characteristics

As shown in Table 1, out of 1047 study participants, 427 children (40.78%) were migrant children. There were notable differences between the two groups of children in terms of age, type of family, number of siblings, parental education, parental presence at home, parental guidance in studies, experience of transferring to another school, having their own room or not, and academic performance.

An extended family was defined as one where children lived with both parents and at least one grandparent, while a nuclear family comprised only the children and their parents. In this study, 60.66% of migrant children belonged to nuclear families, which was much higher than the percentage of those belonging to extended families and other types. Additionally, the frequency of both parents being often absent from home was greater among migrant children compared to urban children, resulting in migrant children receiving less companionship and care from their parents and grandparents. Further, 65.16% of urban children had fathers with a college education or higher and 64.68% had mothers with similar education levels. In contrast, the proportion of migrant children whose parents had an education level of college and above was less than 10%. Only 26.7% of migrant children frequently received study guidance from their parents, compared with 45.32% of urban children. Regarding school transfers, 51.86% of migrant children had experienced at least one school transfer, compared to only 2.58% of urban children.

### 3.2. Comparison of Creative Tendencies

Table 2 shows the results of independent sample *t* tests conducted to examine the differences in creativity across the four dimensions and total scores between urban and migrant children. A Kolmogorov–Smirnov normality test was conducted on the creativity propensity score, with a kurtosis of 0.236 and a skewness of 0.118 (*p* > 0.05). It showed that the effect of migration domicile status (migrant vs. urban) on creativity was significant, with urban children having an advantage over migrant children. The mean score for the full dimension of creativity for urban children was 114.42, compared to 107.86 for migrant children (*p* < 0.001). For the adventurous dimension, urban children had an advantage over migrant children (*p* < 0.001), with a score of 25.36 for urban children compared to 23.74 for mobile children. For the curious dimension, urban children had an advantage over migrant children (*p* < 0.001), with a score of 33.51 for urban children compared to 31.66 for migrant children. For the imaginative dimension, urban children had an advantage over migrant children (*p* < 0.001), with a score of 27.14 for urban children compared to 25.66 for migrant children. For the challenging dimension, urban children had an advantage over migrant children (*p* < 0.001), with a score of 28.41 for urban children compared to 26.80 for migrant children.

### 3.3. Children’s Level of Creativity in Different Grades

Differences in creativity were observed across grade levels (Table 3). While no significant differences in total creativity scores and curiosity scores were detected, there were significant differences in adventurousness, imagination, and willingness to challenge. These dimensions of creativity were significantly higher in fourth graders compared to fifth and sixth graders.

### 3.4. Regression Analyses of Factors Predicting Children’s Creativity

In this study, four explanatory variables were selected according to the “Basic Child Information Questionnaire”, which were household registration status, age and gender, family environment, and school environment. The creativity tendency of all students was regressed on the above factors and models 1–4 were established. According to the results of the multi-model regression analysis (Table 4), the factors affecting migrant children and urban children could be analyzed as follows. We tested for multicollinearity among the independent variables in the model and found no evidence of multicollinearity as VIF values were all below 3.

**Model 1:** The effect of migrant status on children’s creativity was significant. The results of the regression analysis for the whole sample showed that the average score of migrant children on the measure of creativity was 0.26 points lower than that of urban children. This indicates that migrant status has a notable negative impact on children’s creativity.

**Model 2:** The gender and age of children had no significant impact on creativity, while migrant status still had a significant negative impact on children’s creativity, net of these characteristics.

**Model 3:** The variables related to the family environment were added to model 2 in the following order: standard family, only child, good parent–child relationship, higher education of the parents, often being accompanied by the father and/or mother, studies often guided by the parents, and having a room of one’s own. Among these, a good parent–child relationship, parents often directing studies, and having their own room were positively associated with creativity. The inclusion of these environmental factors diminished the effect of migrant status on children’s creativity (b = 0.03). These variables potentially moderate the relationship between migrant status and creativity, indicating that migrant children may face home environments that are less conducive to creativity, such as the absence of a personal room (*p* < 0.001) and infrequent academic guidance from parents (*p* < 0.001).

**Model 4:** The magnitude of the effect of migrant status on children’s creativity was reduced upon the inclusion of school transfers and academic performance. However, migrant status still had a significant negative association with creativity, while a good parent–child relationship, frequent parental guidance in studying, and having one’s own room still had a significant positive association. In addition, transferring to another school had no significant effect on creativity, while good academic performance had a positive effect on creativity.

## 4. Discussion

China is one of the world’s most rapidly urbanizing countries, and this growing urbanization has led to an increasing disparity both within and between its cities. This disparity encourages migration from smaller towns or cities to better-resourced urban areas [25]. In recent years, mobile populations have been increasingly migrating to big cities with their parents to obtain better educational resources and a better quality of life [26]. As such, the educational and psychological aspects of migrant children, among others, are worthy of exploration and attention. This study examines the current status and differences in creativity between migrant and urban children and examines the factors that influence creativity.

This study represents the first of its kind to focus on migrant children in Hangzhou. Hangzhou, as a new first-tier city, has witnessed yearly increases in the migrant population, making it an important context to study. This study utilized the Williams Creative Tendencies Measurement Scale to assess children’s creativity. The scale assesses an individual’s creative potential by examining key personality traits including adventurousness, curiosity, imagination, and willingness to challenge, making it both comprehensive and authoritative. Regarding limitations, this study focused on the impact of family and household dynamics on the creativity levels of migrant and urban children. The school environment aspect only considered the factor of whether or not children had changed schools and did not comprehensively consider other factors that may have an impact on creativity. In addition, the respondents were only selected from two schools in Linping District, Hangzhou, which may be limited in terms of promoting applicability. Future research could take teaching methods, teacher–student relationships, etc., into account.

Even with these limitations, the findings of this study constitute an important contribution to our current body of knowledge regarding migrant children’s well-being and their levels of creativity. The results suggest that urban children score higher than migrant children in creativity overall, as well as its component dimensions. This is consistent with the results of Li Jinzhen and colleagues, who found that urban children’s creative thinking surpasses that of migrant children [27]. The lower creativity of migrant children compared to urban children may be related to inadequate educational resources [28] and mental health issues [29].

The Williams Creative Tendency Measurement Scale shows that migrant children have lower creativity scores than urban children, which may be affected by factors such as mobility [30]. We conducted a regression analysis of the factors considered and found that creativity may be influenced by multiple factors such as mobility, parental guidance in learning, the parent–child relationship, having their own room, and academic performance.

Kandler and colleagues found that individual differences in creativity are influenced by life circumstances and that mobility has an impact on children’s creativity [31]. Migrant children, amidst environmental changes, are faced with challenges such as adapting to life and integrating into society [32]. Such circumstances often lead to lower self-esteem, greater timidity, and higher rates of depression compared to their peers [16]. Research suggests that people with better mental health tend to be more creative [33]. Further, with the economic support of their families, urban children have more opportunities to experience different cultures and broaden their horizons than migrant children. This is important, as research also shows that diversified life experiences can enhance the development of creativity in children [34]. This study compared the creativity of urban children and migrant children, confirming that the influence of mobility factors limits the development of creativity and mobility has a negative impact on children’s creativity. Parents should attach great importance to the psychological and educational problems of children’s adaptation to new life after migration and assist children to adapt to life in a timely manner.

This study not only proves that mobility has a negative effect on children’s creativity but also points out that creativity is influenced by the family environment, which is one of the most important contexts for children’s development. Our results indicate that a good family environment, positive family education, and a harmonious parent–child relationship enable children to gain more self-confidence and strength to develop themselves and improve their creativity, consistent with past research showing the importance of the family context [35].

Results from regression analyses showed that good parent–child relationships are positively related to creativity, consistent with the results of previous research [36]. Positive and engaged interaction between parents and children plays a crucial role in guiding children toward a proper understanding of the world. Such interactions not only foster children’s exploratory enthusiasm but are also instrumental in nurturing creativity.

The comparison of parental guidance in learning revealed that such guidance shapes children’s creativity. Parents of migrant children typically have a lower level of education and are less engaged with, and have lower expectations for, their children’s education. As a result, the amount of time and effort spent on guiding children’s learning is substantially lower, as evidenced by a number of studies [37,38]. Hoferichter and colleagues also found that parental educational support has a profound effect on creativity and that parental attitudes toward education influence the degree to which children value their education [39]. Xinmei found that migrant children’s concentration and engagement in learning were significantly lower than that of non-migrant children, and that engagement in learning had a notable impact on academic performance [40]. The results of this study also showed that academic performance is related to creativity. Children with better academic performance tended to have higher creativity scores.

The results found significant differences in the creativity of children with and without their own room, consistent with past work which found that a child’s housing conditions can influence a child’s physical and mental health, social skills, emotional development and maturity, language and cognitive development, and communication skills [41]. The results of the survey showed that migrant children are less likely than urban children to have a separate room and that most migrant children will live with their parents in overcrowded shared rooms due to economic constraints and the high cost of renting. Research has found that children in crowded home environments perceive themselves as less capable of learning [41], suggesting that relatively independent space enhances the brain’s ability to process information. Thus, it is likely beneficial for parents to respect their children’s privacy and provide them with the space to relax physically and mentally, fostering an environment conducive to the development of creativity.

Conversely, the impact of a child’s gender on creativity is found to be insignificant, suggesting that both males and females possess comparable creative abilities. This observation may be attributed to modern educational philosophies that emphasize equality and inclusiveness, thereby mitigating traditional gender disparities in creative expression.

Variations in creativity scores were also observed among migrant children across different grades, with fourth graders exhibiting higher levels of adventurousness, imagination, and challenge compared to fifth and sixth graders. This trend may be attributed to the greater willingness of younger children to think and act boldly. Further, research by Su Jun found that test-oriented education can hinder the development of creativity [42]. Older students, who are more deeply influenced by test-oriented education and deprived of extracurricular practical activities for long periods of time, tend to lose the motivation to explore and solve problems, resulting in lower creativity. Taken together, this highlights the importance of moving away from traditional teaching models to approaches that encourage problem exploration in a way that provides greater opportunities for creative development.

In general, this study found that mobility has a negative impact on children’s creativity, and the creativity of migrant children is lower than that of urban children, which is consistent with most previous research conclusions. Children’s creativity is affected by the family environment. Having a good parent–child relationship and family atmosphere play a positive role in children’s creativity. The occurrence of transfer events was not found to have an impact on creativity, but good academic performance had a positive impact on creativity. Based on this, attention should be paid at the national level to the education of migrant children to actively solve the problem whereby migrant children are limited by household registration and a lack of good educational resources; the family influences the lifelong education of children and has an important influence on the formation of children’s creativity. Parents should pay attention to children’s psychological development, create a harmonious family atmosphere, and promote the development of children’s creativity in order to better adapt to the needs of future society.

## 5. Conclusions

The principal findings of this study highlight that migrant children exhibit lower levels of creativity compared to urban children, with significant differences observed between the two groups. Factors such as the parent–child relationship, parental guidance, having their own room, and academic performance were identified as consequential to children’s creativity. Future research should examine these relationships in other contexts, with a wider sampling of schools, and include school-level variables in addition to the family and household variables included here.

## Figures and Tables

**Table 1:** Comparison of socio-demographic and family characteristics of migrant and urban children.

	Urban Children n = 620	Migrant Children n = 427	x^2^/t	*p*
Age (M ± SD)	11.06 ± 0.86	10.92 ± 1.01	2.421	<0.05
Sex (n, %)				
Male	333 (53.71)	252 (59.02)	*	0.0996
Female	287 (46.29)	175 (40.98)		
Family Type (n, %)				
Extended family	307 (49.52)	142 (33.26)	29.75	<0.001
Nuclear family	272 (43.87)	259 (60.66)		
Other types	41 (6.61)	26 (6.09)		
Number of siblings (n, %)				
Zero	225 (36.29)	117 (27.40)	41.23	<0.001
One	311 (50.16)	184 (43.09)		
Two	63 (10.16)	90 (21.08)		
Three or more	21 (3.39)	36 (8.43)		
Parent–child relationship (n, %)				
Good	527 (85.00)	373 (87.35)	4.094	0.1291
General	87 (14.03)	48 (11.24)		
Poor	36 (5.81)	36 (8.43)		
Level of education of fathers (n, %)				
Primary and below	36 (5.81)	68 (15.93)	403.4	<0.001
Junior	32 (5.16)	195 (45.67)		
Senior	147 (23.71)	119 (27.87)		
College and above	404 (65.16)	41 (9.60)		
Unknown	1 (0.16)	4 (0.94)		
Level of education of mother (n, %)				
Primary and below	33 (5.32)	85 (19.91)	354.3	<0.001
Junior	63 (10.16)	176 (41.22)		
Senior	123 (19.84)	125 (29.27)		
College and above	401 (64.68)	37 (8.67)		
Unknown	0	4 (0.94)		
Parents at home (n, %)				
Both parents are home a lot	396 (63.87)	234 (54.80)	25.22	<0.001
Father is away a lot	137 (22.10)	100 (23.42)		
Mother is away a lot	25 (4.03)	20 (4.68)		
Both parents are away a lot	38 (6.13)	63 (14.75)		
Father or mother only	24 (3.87)	10 (2.34)		
Parental guidance for learning (n, %)				
Regularly	281 (45.32)	114 (26.70)	47.14	<0.001
Sometimes	262 (42.26)	208 (48.71)		
Rarely	77 (12.42)	105 (24.59)		
Transfer experience (n, %)				
Never	604 (97.42)	206 (48.24)	349.4	<0.001
Once	14 (2.26)	171 (40.05)		
Many times	2 (0.32)	50 (11.71)		
A room of your own (n, %)				
No	62 (10.00)	169 (39.58)	*	<0.001
Yes	558 (90.00)	258 (60.42)		
Academic performance (n, %)				
Excellent	109 (17.58)	64 (14.99)	19.71	<0.05
Good	266 (42.90)	149 (34.89)		
General	224 (36.13)	177 (41.45)		
Poor	21 (3.39)	37 (8.67)		

* Indicates that Fisher’s exact test was used. M ± SD represents Mean ± standard deviation.

**Table 2:** Creativity tendency score for migrant and urban children.

	Urban Children	Migrant Children	t	*p*
Total score (M ± SD)	114.42 ± 11.80	107.86 ± 11.96	8.782	<0.001
Different dimensions (M ± SD)				
Adventurousness	25.36 ± 3.40	23.74 ± 3.09	7.867	<0.001
Curiosity	33.51 ± 4.11	31.66 ± 4.37	6.946	<0.001
Imagination	27.14 ± 4.16	25.66 ± 4.28	5.600	<0.001
Challenge	28.41 ± 3.06	26.80 ± 3.39	8.000	<0.001

M ± SD represents Mean ± standard deviation.

**Table 3:** Creativity tendency scores of migrant children in different grades.

	Fourth Grade	Fifth Grade	Sixth Grade	F	*p*
Total score (M ± SD)	110.31 ± 11.85	107.07 ± 11.73	107.14 ± 12.06	2.787	0.0627
Different dimensions (M ± SD)					
Adventurousness	24.14 ± 3.25	23.75 ± 2.90	23.47 ± 3.15	472.6	<0.001
Curiosity	32.01 ± 4.40	31.27 ± 4.46	31.86 ± 4.22	1.149	0.3180
Imagination	26.62 ± 4.19	25.31 ± 4.19	25.42 ± 4.34	3.401	<0.05
Challenge	27.53 ± 3.67	26.73 ± 3.25	26.40 ± 3.28	3.565	<0.05

M ± SD represents Mean ± standard deviation.

**Table 4:** Multiple regression analysis of factors influencing children’s creativity.

Items	Model 1		Model 2		Model 3		Model 4	
	Registered Residence Status	VIF	Age and Gander	VIF	Family Environments	VIF	School Environments	VIF
Variable	β (P)		β (P)		β (P)		β (P)	
Registered residence status								
Migrant	−0.261 (<0.001)	1.000	−0.264 (<0.001)	1.008	−0.231 (<0.001)	1.702	−0.208 (<0.001)	2.190
Demographic characteristics								
Age			−0.003 (0.662)	1.010	−0.008 (0.791)	1.081	−0.007 (0.83)	1.082
Sex			0.042 (0.161)	1.006	0.048 (0.111)	1.021	0.044 (0.136)	1.027
Family environment								
Standard family					0.04 (0.215)	1.172	0.028 (0.371)	1.177
Only child					−0.009 (0.76)	1.083	−0.01 (0.731)	1.084
Good parent–child relationship					0.081 (<0.05)	1.052	0.08 (<0.05)	1.052
Father received higher education					0.007(0.867)	2.289	0.008 (0.85)	2.291
Mother received higher education					−0.029 (0.520)	2.310	−0.049 (0.271)	2.333
Parental companionship					−0.039 (0.228)	1.052	−0.047 (0.15)	1.229
Parental guidance in learning					0.124 (<0.001)	1.098	0.112 (<0.001)	1.105
Having one’s own room					0.088 (<0.05)	1.171	0.093 (<0.05)	1.172
School environment								
Never transferred to another school							0.052 (0.15)	1.532
Excellent academic performance							0.114 (<0.001)	1.032

VIF represents variance inflation factor.

## Data Availability

All data generated or analyzed during this study are included in this published article.

## References

[1] Sternberg R.J. (2005). Handbook of Creativity.

[2] Alfonso-Benlliure V., Meléndez J.C., García-Ballesteros M. (2013). Evaluation of a creativity intervention program for preschoolers. Think. Ski. Creat..

[3] Runco M.A., Millar G., Acar S., Cramond B. (2010). Torrance Tests of Creative Thinking as Predictors of Personal and Public Achievement: A Fifty-Year Follow-Up. Creat. Res. J..

[4] Yang Y. (2023). Research on the Level of Creativity Development of Preschool Children and Its Influencing Factors. Master’s Thesis.

[5] Wang X., Shen J., Shi B. Differences in influencing factors of creativity between rural and urban children. Proceedings of the Summary of Papers from the 2006 Academic Annual Meeting of the Development Psychology Professional Committee of the Chinese Psychological Society and the Education Psychology Professional Committee of the Chinese Psychological Society.

[6] Han Q., Hu W. (2005). A Study on the Development of Creative Literature Problem posing Ability in Primary School Students. Psychol. Dev. Educ..

[7] Liu G., Shi J. (2004). Development and Education of Creative Thinking among Township Primary and Secondary School Students. Chin. J. Spec. Educ..

[8] Shi B., Qian M., Lu Y., Plucker J.A., Lin C. (2012). The Relationship Between Migration and Chinese Children’s Divergent Thinking. Psychol. Aesthet. Creat. Arts.

[9] Shi B., Lu Y., Dai D.Y., Lin C. (2013). Relationships Between Migration to Urban Settings and Children’s Creative Inclinations. Creat. Res. J..

[10] Fang W. (2014). A Study of Creative Self-Efficacy and Creative Thinking in Migrant Children. Master’s Thesis.

[11] Cheng L., Wang Y., He C., Duan D., Pang Y. (2017). A comparative study on the creative development of mobile and urban gifted children. Chin. J. Spec. Educ..

[12] Wang Y., He C., Cheng L. (2017). A comparison of creative development between mobile gifted children aged 12–13 and urban gifted children. J. Shangrao Norm. Univ..

[13] Jiang Z., Shi C., Lv L. The relationship between educational placement methods and positive psychological development of migrant children. Proceedings of the Annual Meeting of the Chinese Society of Social Psychology and Cultural Psychology Summit Forum, Hubei Provincial Psychology Association Annual Meeting.

[14] Shen J., Wang X. (2007). What Does Mobility Mean for Children—A Rethinking of a Psychological Study. Educational Exploration 2006. Chinese Women’s Movement.

[15] Sternberg R.J., Lubart T.I. (1995). Defying the Crowd: Cultivating Creativity in a Culture of Conformity. Handbook of Industrial and Organizational Psychology Volume 4.

[16] Shuang M., Yiqing W., Ling J., Guanzhen O., Jing G., Zhiyong Q., Xiaohua W. (2022). Relationship between parent-child attachment and depression among migrant children and left-behind children in China. Public Health.

[17] Karina H.K., Vanessa A.F., Laura O. (2024). Socioeconomic status effects on children’s creativity: The mediating role of executive functions. Think. Ski. Creat..

[18] Jiang H., Su Y., Zhuang R. (2024). Experimental Study on the Influence of Different Emotions on the Creativity of Children and Adolescents. Med. Inf..

[19] Xie R. (2023). The Impact of Art Education on the Development of Children’s Creativity. Delta.

[20] Jiang H., Su Y., Zhuang R., Ning Y., Wang G., Lei Z., Zhang Y. (2012). The Research of Migrant Children’ Characteristic of Creative Thinking and Training. 2011. Master’s Thesis.

[21] Xu S., Li D., Du M. (2019). A study on the influencing factors of enhancing children’s creativity in the new western region. New West..

[22] Shi B., Xu L., Xu J. (2009). The relationship between happiness, security, and social exclusion among migrant children. Psychol. Sci..

[23] Yin H., Wu N. (2013). A comparative study on the differences between migrant children and urban children in primary and secondary school mental health education. Ment. Health Educ. Prim. Second. Sch..

[24] Lin H., Wang M. (1997). Williams Creativity Assessment Packet.

[25] Gong J., Rao N. (2023). Preschool experiences and home learning environments of migrant children in urban China. Front. Public Health.

[26] Xu H., Pan J., Ren Z., Tetsu-Suen S.Y. (2024). Overview, Hot Spots and Trends of Domestic Research on Migrant Children—An Exploration Based on Hot Spots Visualization. J. Suzhou Univ. Sci. Technol. (Soc. Sci. Ed.).

[27] Li J., Wang W., Shi J. (2004). Development of practical creativity in children and its relationship to the family environment. Acta Psychol. Sin..

[28] He S., Miao C. (2023). Study on the Education of Urban Migrant Children from the Perspective of Developmental Social Policies. Educ. Rev..

[29] Wan Z. (2020). A bibliometric analysis of 20 years of research on the mental health of migrant children. Psychol. Res..

[30] Liu W., Li M. (2010). New developments in the study of children’s creative personality. J. Educ. Sci. Hunan Norm. Univ..

[31] Kandler C., Riemann R., Angleitner A., Spinath F.M., Borkenau P., Penke L. (2016). The nature of creativity: The roles of genetic factors, personality traits, cognitive abilities, and environmental sources. J. Personal. Soc. Psychol..

[32] Chen X., Li D., Xu X., Liu J., Fu R., Cui L., Liu S. (2019). School adjustment of children from rural migrant families in urban China. J. Sch. Psychol..

[33] Yu G., Zhang W. (2020). Creativity and Mental Health: Controversies, Evidence, and Perspectives on Research. Hebei J..

[34] Shao Y., Zhang C., Zhou J., Gu T., Yuan Y. (2019). How Does Culture Shape Creativity? A Mini-Review. Front. Psychol..

[35] Zhang J., Zheng L. (2018). Analysis of the influencing factors of parent-child relationship on children’s creativity development. Mudanjiang Norm. Univ. (Philos. Soc. Sci.).

[36] Li A., Zhang J. (2020). The prediction of parent-child relationship and empathy on adolescents’ social creativity: The mediating role of friendship quality. Psychol. Behav. Res..

[37] Lü C., Yang P., Sun J., Ming Y. (2023). The relationship between family socioeconomic status and their academic achievement among county high school students: A chain-mediated model based on parental educational involvement and self-educational expectations. Chin. J. Health Psychol..

[38] Yu X. (2020). Parents’ social background, educational values and their educational expectations. Nanjing Norm. Univ. (Soc. Sci. Ed)..

[39] Hoferichter F., Kulakow S., Hufenbach M.C. (2021). Support from Parents, Peers, and Teachers Is Differently Associated with Middle School Students’ Well-Being. Front. Psychol..

[40] Cao X. (2021). The impact of migrant children’s engagement in learning on learning satisfaction: The mediating role of academic performance. Spec. Educ. China.

[41] Evans G.W. (2006). Child development and the physical environment. Annu. Rev. Psychol..

[42] Su J. (2010). Exam-oriented education hinders creativity. The higher the grade, the lower the creativity. Road Achiev..

